# End-of-life care values and treatment preferences among Japanese breast cancer patients and families: A dyadic cross-sectional study

**DOI:** 10.1016/j.breast.2026.104899

**Published:** 2026-08-12

**Authors:** Akemi Hara, Ayu Ajitomi, Kazunoshin Tachibana, Kenji Gonda, Masahiro Wada, Rui Omori, Megumi Arai, Kayoko Konuma, Toyoaki Sawano, Yoshiaki Kanemoto, Hirotomo Miyatake, Tomohiro Kurokawa, Yukiko Kouchi, Norio Kanzaki, Yasuhiro Kotera, Yoshitake Takebayashi, Michio Murakami, Tohru Ohtake, Hiroaki Shimmura, Akihiko Ozaki

**Affiliations:** a Clinical Research Center, Jyoban Hospital of Tokiwa Foundation, Iwaki, Japan; b Department of Biostatistics, The University of North Carolina at Chapel Hill, Chapel Hill, North Cartolina, USA; c Breast and Thyroid Center, Jyoban Hospital of Tokiwa Foundation, Iwaki, Japan; d Department of Breast Surgery, Fukushima Medical University School of Medicine, Fukushima, Japan; e Department of Breast Surgery, Utsunomiya Central Clinic, Utsunomiya, Japan; f Department of Breast Cancer, St. Luke's International Hospital, Tokyo, Japan; g Department of Nursing, Jyoban Hospital of Tokiwa Foundation, Iwaki, Japan; h Department of Surgery, Jyoban Hospital of Tokiwa Foundation, Iwaki, Japan; i Yushoukai Medical Corporation Nobishiroclinic, Fujisawa, Japan; j Department of Urology, Jyoban Hospital of Tokiwa Foundation, Iwaki, Japan; k School of Health Sciences, Institute of Mental Health, University of Nottingham, Nottingham, United Kingdom; l Department of Health Risk Communication, Fukushima Medical University School of Medicine, Fukushima, Japan; m Center for Infectious Disease Education and Research, The University of Osaka, Suita, Japan; n EIPM Center, The University of Osaka, Suita, Japan

**Keywords:** Advance care planning, Breast neoplasms, Family relationships, Patient preference, Decision making

## Abstract

**Background:**

Patient–family discordance in end-of-life values complicates advance care planning (ACP) but is rarely quantified in Asian oncology settings. We measured patient–family differences in values and treatment preferences in breast cancer and correlates of discordance.

**Methods:**

In this single-center cross-sectional study at a Japanese breast-cancer unit (August 2024–May 2025), 470 patients completed a questionnaire on Life Priorities and Values (14 items) and End-of-Life Care Preferences (6 items); 218 were matched to 324 family respondents. Analyses used generalized linear mixed-effects models, Cohen's κ, multiple correspondence analysis (MCA), an actor–partner interdependence model (APIM) on MCA dimension scores, and a total discordance score.

**Results:**

Patients prioritized autonomy (“Independence in ADL,” OR = 0.20; “Not being a burden to family,” OR = 0.31), families intervention (“Longevity,” OR = 2.15; “Treatment until satisfaction,” OR = 4.50; all p < .001). Concordance was uniformly poor (κ 0.03–0.32) and directional: 88.5% of disagreements on treatment pursuit were on the family-endorsement side. Prior ACP discussion was associated with lower discordance (median 6 vs 7, p < .001), chiefly for palliative items, and chemotherapy with higher discordance (p = .012). Exploratory APIM showed strong actor associations and two partner associations for family Preference Dim2: patients’ greater value engagement and longevity orientation were each associated with higher family endorsement of active treatment (p = .022 and 0.002).

**Conclusions:**

Systematic, directional patient–family discordance was present across nearly all end-of-life domains. Prior ACP discussion was associated with lower discordance, particularly for palliative preferences, supporting structured dyadic communication in breast cancer care.

## Highlights

• Breast cancer patients and their families disagreed on most end-of-life values.
• Discordance was directional: families wanted more treatment than patients chose.
• 88.5% of treatment-pursuit disagreements fell on the family-endorsement side.
• Prior patient-family discussion was associated with lower total discordance.
• Chemotherapy receipt was associated with greater patient-family discordance.

## Graphical abstract

**Figure:**
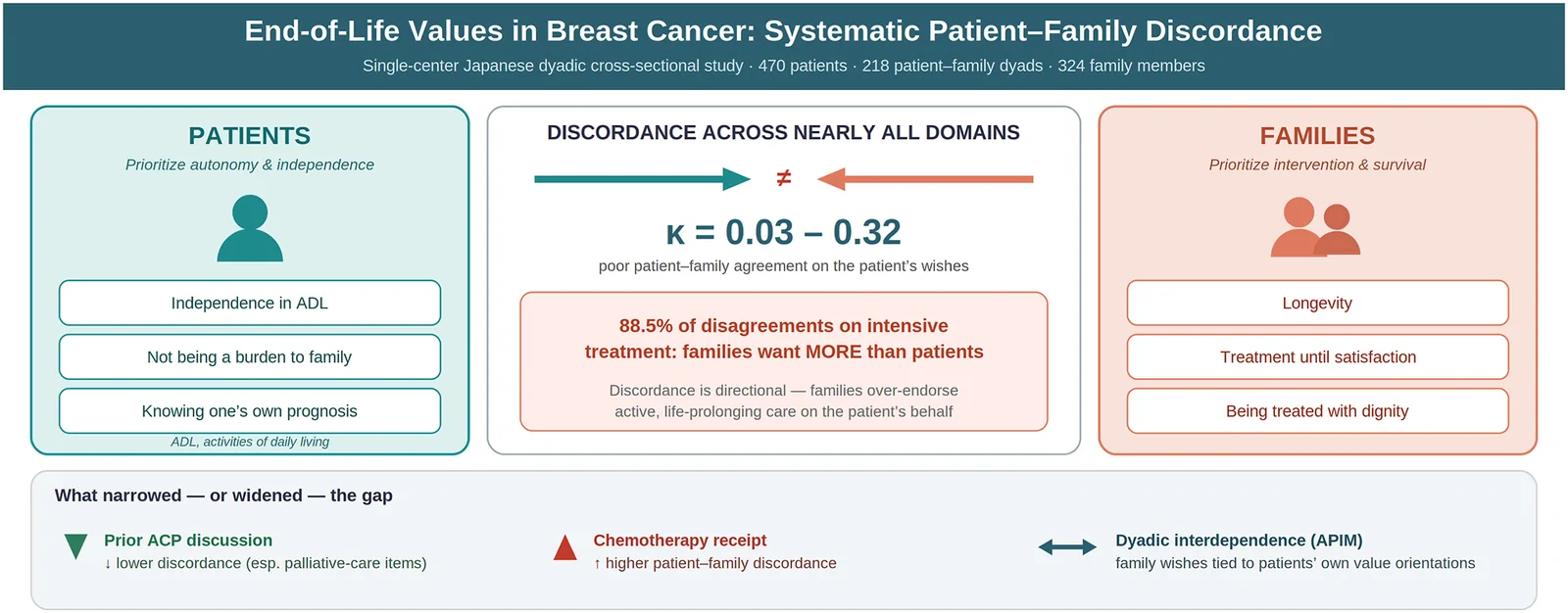

## 1 Background

Advance care planning (ACP) supports care aligned with patients' values, goals, and preferences, and is increasingly emphasized in oncology [1,2]. For breast cancer patients, decisions often extend across surgery, endocrine therapy, chemotherapy, survivorship, recurrence risk, and uncertain prognosis, making early preference-sensitive communication important [3,4]. ACP has been associated with better patient satisfaction, less psychological distress, and more appropriate resource use; for families, it may reduce decisional regret and improve confidence when surrogate decisions are required [5,6]. Nevertheless, family members do not always understand patients' wishes. Systematic reviews and serious-illness studies show that surrogates often misjudge treatment preferences, [7,8] and families may overestimate life-prolonging preferences while underestimating comfort-focused goals [9,10]. In Japan, ACP implementation is further complicated by short consultation times, fee-for-service pressure on outpatient encounters, and cultural patterns in which family preferences may carry substantial weight when values are misaligned [11,12].

Prior ACP frameworks distinguish broad life priorities from specific treatment preferences, [13,14] while intervention and surrogate-preparation studies emphasize dyadic communication and the need to prepare spokespersons before crisis decisions arise [[15], [16], [17], [18]]. However, ACP studies in oncology rarely combine four levels of evidence in the same cohort: item-level patient–family comparison, dyad-level concordance, clinical predictors of discordance, and dimension-level interdependence between patient and family responses.

This exploratory cross-sectional study therefore compared end-of-life values and treatment preferences between Japanese breast cancer patients and family members; quantified concordance, discordance direction, and total discordance burden; examined associations with ACP discussion, clinical characteristics, and surrogate designation; and explored actor–partner associations between MCA-derived value orientations and treatment preferences. A methodological distinction is important for interpreting these aims. The family questionnaire did not ask family members to report their own life values: for the value items it asked them to predict what the patient considered important, whereas for the treatment-preference items it asked what care they themselves would want the patient to receive. Family value responses are therefore predictions about the patient, and family preference responses are the family's own choices made on the patient's behalf.

## 2 Methods

### 2.1 Study design and setting

This single-center cross-sectional study analyzed questionnaire data collected at the Breast and Thyroid Center of Joban Hospital, Tokiwa Foundation. Clinical and treatment variables were extracted from medical records for all patients, including clinical stage, breast cancer subtype, menopausal status, breast cancer family history, curative surgery, hormone therapy, chemotherapy or targeted therapy, and timing of questionnaire completion relative to curative surgery. The reporting adheres to the STROBE guidelines.

### 2.2 Survey instruments

The ACP questionnaire was developed internally for this study. No existing validated Japanese instrument covered both broad life priorities and specific end-of-life care preferences in a format short enough for routine breast cancer outpatient use, so items were generated de novo rather than adapted from a published scale. Item development proceeded in three steps. First, a candidate item pool was drafted from a review of the ACP and end-of-life care literature, drawing on established frameworks that separate broad life priorities from specific treatment preferences and on the Japanese Ministry of Health, Labour and Welfare guidelines on the decision-making process for end-of-life care [1,13,14]. Second, the pool was reviewed in iterative discussions among the clinical staff of the Breast and Thyroid Center, including breast surgeons, oncology nurses, and clinical research staff, who assessed each candidate item for clinical relevance, comprehensibility to patients without further explanation, and cultural appropriateness; items judged redundant, ambiguous, or unlikely to be understood within a short outpatient encounter were reworded or dropped by consensus. Third, the retained items were arranged into the two analytic domains and the wording was finalized by consensus among the study clinicians before data collection began. Fourth, a feasibility check was carried out during early data collection: responses from an initial batch of participants were reviewed to confirm that the items were useable in practice and to inspect the response distribution of each item, and this review informed which items were retained in the final analytic set. Content validity therefore rests on literature grounding, clinical consensus, and this feasibility review. No formal cognitive interviewing was conducted and no psychometric reliability or factor-analytic study was performed, so the instrument has not been psychometrically validated.

The final instrument covered two analytic domains: Life Priorities and Values (14 binary items) and End-of-Life Care Preferences (6 analyzed binary items). Two further item-selection steps were applied at the analysis stage. Two institutional-care items, treatment in a hospital and treatment in a residential facility, were combined into a single facility/hospital treatment variable because they addressed the same underlying construct of receiving care outside the home. Three preference items phrased as the logical opposites of retained items were excluded to avoid redundancy, since endorsement of one necessarily implied non-endorsement of its counterpart. “Prognostic awareness” referred to the patient's wish to know prognosis, including life expectancy, likelihood of cure, and disease trajectory.

Throughout this paper, family, family member, and family respondent denote any person whom the patient herself named as someone who could convey her wishes on her behalf and who returned a family questionnaire, irrespective of biological or legal relationship; the category is defined by patient nomination rather than by kinship and therefore includes friends and partners as well as relatives. Patients answered about their own values and preferences. The family questionnaire used parallel item wording but differed in what it asked by domain, and this difference matters for interpretation. For the 14 value items, family members were asked to predict what the patient considered important; those responses are therefore family predictions of patient values, not the family's own personal values. For the 6 preference items, family members were asked what treatment or care they would want the patient to receive if the patient became severely ill; those responses are the family's own preferences expressed on the patient's behalf. The patient questionnaire also asked respondents to identify a preferred surrogate decision-maker.

The family questionnaire additionally asked whether the respondent had ever talked with the patient about these topics, with response options of “have discussed,” “have not discussed,” and “do not wish to discuss.” Throughout this paper, prior ACP discussion denotes an affirmative answer to that single item. It therefore refers to any previous conversation between the family respondent and the patient about the end-of-life topics covered by the questionnaire, reported by the family member. It does not denote a structured or clinician-facilitated ACP session, a documented goals-of-care discussion, or the existence of an advance directive or other written directive; none of these were recorded in this study, and no information was collected on when the conversation occurred, how detailed it was, or whether the patient agreed that it had taken place.

### 2.3 Participants and recruitment

Between August 1, 2024, and May 31, 2025, questionnaires were administered during routine outpatient care to breast cancer patients judged by the attending physician to have decision-making capacity. Completion constituted consent; patients could complete the questionnaire in clinic or at home, and family assistance with completion was permitted when needed. The patient questionnaire asked each patient to name one or more trusted people who could convey her wishes on her behalf if she were no longer able to do so — the surrogate decision-maker item — with pre-specified response options of mother, father, sibling, spouse, child, cousin, grandparent, friend, and other; a separate item recorded whether the patient had actually asked those people to speak for her. Eligibility for the family questionnaire followed directly from this item: when a patient named at least one such person, a family questionnaire was provided for each named person and collected either the same day or at a subsequent visit. No restriction was placed on the legal or biological relationship of the named person, so designated surrogates were not necessarily spouses or legal next of kin, and patients could name more than one person; among matched patients, 79 (36.2%) named more than one relationship category. Family membership in this study was therefore operationalized by the patient's own nomination rather than by kinship, and friends and partners named in this way are counted as family respondents throughout.

The initial cohort comprised 470 patients. The primary dyadic cohort included 218 patients with at least one returned family questionnaire, linked to 324 family respondents; 124 patients (56.9%) had one family respondent, 82 (37.6%) had two, and 12 (5.5%) had three or more. The relationship of each family respondent to the patient was recorded in a free-text field and categorized post hoc: child in 142 (43.8%), spouse in 115 (35.5%), sibling in 25 (7.7%), friend or partner in 14 (4.3%), parent in 10 (3.1%), child-in-law or other in-law in 6 (1.9%), niece, nephew, or cousin in 4 (1.2%), grandchild in 1 (0.3%), and other or not recorded in 7 (2.2%). The remaining 252 unmatched patients were excluded from dyadic analyses but retained for descriptive and selection-bias comparisons.

### 2.4 Statistical analysis

Descriptive statistics were calculated for all patients, matched patients, unmatched patients, and family members (Table 1). Missing ACP item data were minimal (≤5 cells, <0.1%); the surrogate designation item "Children" had 5 missing values, coded as not selected, and inferential analyses used item-level listwise deletion. Matched and unmatched patients were compared on all ACP items and surrogate designations using chi-square tests with Cramér's V (Supplementary Table 1).

**Table 1:** Clinical and demographic characteristics of participants.

Item	All Patients (N = 470)	Matched Patients (n = 218)	Unmatched Patients (n = 252)	Family Members (n = 324)
**Demographics**				
Age, years, mean ± SD	62.4 ± 13.7	64.2 ± 13.2	60.9 ± 13.9	52.5 ± 15.5
**Menopausal status**				
Pre-menopausal	148 (31.5%)	56 (25.7%)	92 (36.5%)	—
Post-menopausal	305 (64.9%)	156 (71.6%)	149 (59.1%)	—
Unknown	17 (3.6%)	6 (2.8%)	11 (4.4%)	—
**Clinical stage**				
Stage 0	32 (6.8%)	17 (7.8%)	15 (6.0%)	—
Stage I	209 (44.5%)	101 (46.3%)	108 (42.9%)	—
Stage II	161 (34.3%)	61 (28.0%)	100 (39.7%)	—
Stage III	25 (5.3%)	13 (6.0%)	12 (4.8%)	—
Stage IV	27 (5.7%)	19 (8.7%)	8 (3.2%)	—
Bilateral	1 (0.2%)	1 (0.5%)	0 (0.0%)	—
Unknown	15 (3.2%)	6 (2.8%)	9 (3.6%)	—
**Subtype**				
HR+/HER2+	35 (7.4%)	19 (8.7%)	16 (6.3%)	—
HR+/HER2-	334 (71.1%)	156 (71.6%)	178 (70.6%)	—
HR-/HER2+	29 (6.2%)	13 (6.0%)	16 (6.3%)	—
HR-/HER2-	32 (6.8%)	11 (5.0%)	21 (8.3%)	—
Unknown	40 (8.5%)	19 (8.7%)	21 (8.3%)	—
**Treatment history**				
Breast cancer family history: Yes	97 (20.6%)	37 (17.0%)	60 (23.8%)	—
Curative surgery performed	418 (88.9%)	191 (87.6%)	227 (90.1%)	—
Hormone therapy	326 (69.4%)	158 (72.5%)	168 (66.7%)	—
Chemotherapy/targeted therapy	222 (47.2%)	107 (49.1%)	115 (45.6%)	—
ACP completed post-surgery	401 (85.3%)	183 (83.9%)	218 (86.5%)	—
ACP completed pre-surgery	18 (3.8%)	9 (4.1%)	9 (3.6%)	—
Days from surgery to ACP, median (IQR)	943.0 (442.0-1477.0)	883.5 (453.0-1450.5)	991.0 (430.5-1513.5)	—
**Family respondents per patient**				
1 respondent	—	124 (56.9%)	—	—
2 respondents	—	82 (37.6%)	—	—
≥ 3 respondents	—	12 (5.5%)	—	—
**Life Priorities and Values**				
Engagement in enjoyable and meaningful activities	334 (71.1%)	156 (71.6%)	178 (70.6%)	254 (78.4%)
Fulfillment of social roles	259 (55.1%)	110 (50.5%)	149 (59.1%)	117 (36.1%)
Spending sufficient time with family and friends	332 (70.6%)	152 (69.7%)	180 (71.4%)	237 (73.1%)
Independence in ADL	403 (85.7%)	192 (88.1%)	211 (83.7%)	211 (65.1%)
Longevity/Prolongation of life	171 (36.4%)	81 (37.2%)	90 (35.7%)	167 (51.5%)
Support from religious or spiritual beliefs	27 (5.7%)	15 (6.9%)	12 (4.8%)	29 (9.0%)
Financial stability	247 (52.6%)	105 (48.2%)	142 (56.3%)	151 (46.6%)
Receiving all possible medical treatments	217 (46.2%)	109 (50.0%)	108 (42.9%)	181 (55.9%)
Being treated with dignity and respect	216 (46.0%)	97 (44.5%)	119 (47.2%)	191 (59.0%)
Not being a burden to others	340 (72.3%)	164 (75.2%)	176 (69.8%)	190 (58.6%)
Not being a burden to family	359 (76.4%)	162 (74.3%)	197 (78.2%)	169 (52.2%)
Maintaining a strong appearance to others	101 (21.5%)	48 (22.0%)	53 (21.0%)	63 (19.4%)
Communicating important matters to loved ones	245 (52.1%)	107 (49.1%)	138 (54.8%)	157 (48.5%)
Freedom from pain and suffering	316 (67.2%)	152 (69.7%)	164 (65.1%)	239 (73.8%)
**End-of-Life Care Preferences**				
Prognostic awareness	357 (76.0%)	168 (77.1%)	189 (75.0%)	187 (57.7%)
Receiving treatment until personal satisfaction is met	140 (29.8%)	65 (29.8%)	75 (29.8%)	177 (54.6%)
Prioritizing palliation over prolonging life	328 (69.8%)	160 (73.4%)	168 (66.7%)	232 (71.6%)
Maintaining preferred lifestyle and daily routines	75 (16.0%)	38 (17.4%)	37 (14.7%)	67 (20.7%)
Trust in one's physician	279 (59.4%)	140 (64.2%)	139 (55.2%)	182 (56.2%)
Willingness to receive treatment in a facility	160 (34.0%)	87 (39.9%)	73 (29.0%)	90 (27.8%)
**Surrogate decision-maker**				
Mother	25 (5.3%)	8 (3.7%)	17 (6.7%)	—
Father	7 (1.5%)	1 (0.5%)	6 (2.4%)	—
Siblings	63 (13.4%)	31 (14.2%)	32 (12.7%)	—
Spouse	239 (50.9%)	116 (53.2%)	123 (48.8%)	—
Children	223 (47.4%)	114 (52.3%)	109 (43.3%)	—
Cousin	5 (1.1%)	2 (0.9%)	3 (1.2%)	—
Grandparents	0 (0.0%)	0 (0.0%)	0 (0.0%)	—
Friend	27 (5.7%)	18 (8.3%)	9 (3.6%)	—
**ACP discussion status**				
Discussed	—	—	—	157 (48.5%)
Not discussed	—	—	—	125 (38.6%)
Unwilling	—	—	—	8 (2.5%)^a^

a *Percentages do not sum to 100% because 34 family members (10.4%) did not respond to this item*.

Patient-family differences across the 20 ACP items were estimated with generalized linear mixed-effects models using a logit link and patient-level random intercepts, reported as odds ratios (ORs), 95% CIs, p-values, and intraclass correlation coefficients (Table 2). To examine whether patient-designated surrogate type was associated with item-level discordance, the same models were repeated after stratification by spouse designation and child designation, with Bonferroni-adjusted p-values (Table 3; Supplementary Table 2).

**Table 2:** Patient–family comparison: GLMM results (N = 218 dyads).

Section	Item	Patient n (%)	Family n (%)	OR [95% CI]	p	ICC
**Life Priorities and Values**	Engagement in enjoyable and meaningful activities	156 (71.6%)	254 (78.4%)	1.46 [0.96, 2.23]	0.075	0.16
	Fulfillment of social roles	110 (50.5%)	117 (36.1%)	0.50 [0.34, 0.74]	< 0.001	0.17
	Spending sufficient time with family and friends	152 (69.7%)	237 (73.1%)	1.23 [0.80, 1.89]	0.35	0.3
	Independence in ADL	192 (88.1%)	211 (65.1%)	0.20 [0.12, 0.34]	< 0.001	0.28
	Longevity/Prolongation of life	81 (37.2%)	167 (51.5%)	2.15 [1.43, 3.24]	< 0.001	0.28
	Support from religious or spiritual beliefs	15 (6.9%)	29 (9.0%)	1.67 [0.52, 5.38]	0.393	0.96
	Financial stability	105 (48.2%)	151 (46.6%)	0.94 [0.64, 1.36]	0.735	0.18
	Receiving all possible medical treatments	109 (50.0%)	181 (55.9%)	1.34 [0.90, 1.99]	0.151	0.3
	Being treated with dignity and respect	97 (44.5%)	191 (59.0%)	1.90 [1.31, 2.76]	< 0.001	0.12
	Not being a burden to others	164 (75.2%)	190 (58.6%)	0.37 [0.24, 0.59]	< 0.001	0.33
	Not being a burden to family	162 (74.3%)	169 (52.2%)	0.31 [0.20, 0.48]	< 0.001	0.22
	Maintaining a strong appearance to others	48 (22.0%)	63 (19.4%)	0.83 [0.52, 1.32]	0.43	0.3
	Communicating important matters to loved ones	107 (49.1%)	157 (48.5%)	0.95 [0.64, 1.41]	0.797	0.29
	Freedom from pain and suffering	152 (69.7%)	239 (73.8%)	1.27 [0.84, 1.91]	0.261	0.19
**End-of-Life Care Preferences**	Prognostic awareness	168 (77.1%)	187 (57.7%)	0.32 [0.20, 0.51]	< 0.001	0.25
	Receiving treatment until personal satisfaction is met	65 (29.8%)	177 (54.6%)	4.50 [2.75, 7.37]	< 0.001	0.4
	Prioritizing palliation over prolonging life	160 (73.4%)	232 (71.6%)	0.89 [0.59, 1.36]	0.599	0.19
	Maintaining preferred lifestyle and daily routines	38 (17.4%)	67 (20.7%)	1.27 [0.78, 2.08]	0.342	0.34
	Trust in one's physician	140 (64.2%)	182 (56.2%)	0.67 [0.46, 0.99]	0.042	0.17
	Willingness to receive treatment in a facility	87 (39.9%)	90 (27.8%)	0.50 [0.33, 0.77]	0.001	0.3

**Table 3:** Subgroup GLMM by surrogate designation (Bonferroni-adjusted)^a^

**Item**	Spouse designation		Child designation	
	**Yes (n = 116)**	**No (n = 102)**	**Yes (n = 114)**	**No (n = 104)**
**Life Priorities and Values (14 items)**				
Engagement in enjoyable and meaningful activities	1.55 [0.84, 2.85]	1.39 [0.78, 2.48]	1.77 [1.00, 3.14]	1.19 [0.64, 2.21]
Fulfillment of social roles	0.53 [0.31, 0.89]*	0.48 [0.27, 0.85]*	0.37 [0.22, 0.62]***	0.76 [0.43, 1.36]
Spending sufficient time with family and friends	1.03 [0.56, 1.90]	1.46 [0.79, 2.71]	1.41 [0.79, 2.53]	1.07 [0.56, 2.02]
Independence in ADL	0.18 [0.09, 0.38]***	0.22 [0.10, 0.49]***	0.19 [0.09, 0.41]***	0.21 [0.10, 0.44]***
Longevity/Prolongation of life	2.02 [1.18, 3.47]*	2.34 [1.23, 4.46]*	2.61 [1.44, 4.72]**	1.81 [1.02, 3.23]
Support from religious or spiritual beliefs	1.44 [0.33, 6.22]	2.15 [0.31, 15.06]	2.13 [0.39, 11.63]	1.34 [0.27, 6.73]
Financial stability	1.43 [0.86, 2.39]	0.56 [0.31, 0.99]	0.74 [0.44, 1.25]	1.25 [0.72, 2.17]
Receiving all possible medical treatments	1.42 [0.81, 2.49]	1.25 [0.72, 2.17]	1.13 [0.66, 1.94]	1.67 [0.93, 3.01]
Being treated with dignity and respect	1.92 [1.16, 3.17]*	1.86 [1.07, 3.25]	1.56 [0.95, 2.56]	2.46 [1.38, 4.39]**
Not being a burden to others	0.39 [0.22, 0.70]**	0.35 [0.17, 0.72]**	0.32 [0.17, 0.61]***	0.44 [0.23, 0.84]*
Not being a burden to family	0.25 [0.14, 0.45]***	0.41 [0.21, 0.79]*	0.26 [0.14, 0.48]***	0.37 [0.20, 0.68]**
Maintaining a strong appearance to others	1.36 [0.65, 2.83]	0.57 [0.30, 1.06]	0.82 [0.45, 1.48]	0.80 [0.37, 1.73]
Communicating important matters to loved ones	0.79 [0.46, 1.37]	1.16 [0.65, 2.06]	0.80 [0.47, 1.37]	1.15 [0.64, 2.05]
Freedom from pain and suffering	1.77 [0.99, 3.16]	0.89 [0.49, 1.62]	1.01 [0.58, 1.74]	1.75 [0.92, 3.35]
**End-of-Life Care Preferences (6 items)**				
Prognostic awareness	0.24 [0.12, 0.47]***	0.40 [0.21, 0.75]**	0.27 [0.14, 0.51]***	0.41 [0.21, 0.79]*
Receiving treatment until personal satisfaction is met	7.18 [3.35, 15.37]***	2.89 [1.50, 5.57]**	4.44 [2.29, 8.60]***	4.67 [2.21, 9.89]***
Prioritizing palliation over prolonging life	1.36 [0.80, 2.33]	0.47 [0.23, 0.95]	1.00 [0.57, 1.74]	0.75 [0.40, 1.41]
Maintaining preferred lifestyle and daily routines	1.21 [0.60, 2.46]	1.33 [0.67, 2.65]	1.42 [0.71, 2.85]	1.16 [0.57, 2.33]
Trust in one's physician	0.73 [0.43, 1.22]	0.61 [0.34, 1.08]	0.51 [0.30, 0.89]*	0.90 [0.52, 1.56]
Willingness to receive treatment in a facility	0.35 [0.18, 0.67]**	0.67 [0.38, 1.20]	0.40 [0.23, 0.70]**	0.67 [0.34, 1.33]

a OR [95% CI] from GLMM with patient-level random intercept; OR > 1 = higher family endorsement. Bonferroni-adjusted (×2) within each surrogate type. *p < .05, **p < .01, ***p < .001.

Dyad-level agreement was summarized using concordance, Cohen's κ with 95% CIs, and McNemar's test for each item (Table 4). For patients with multiple family respondents, family-any endorsement was used so that each patient contributed one dyad-level family composite. Discordance direction was classified as Patient+/Family− or Patient−/Family+, and total discordance score was defined as the number of discordant items from 0 to 20. Associations between discordance and ACP discussion status were examined at item and total-score levels (Table 5). Associations between total discordance score and clinical factors, including disease stage, chemotherapy, hormone therapy, ACP timing, days from surgery to ACP, and patient age, were tested using Mann-Whitney U tests or Spearman correlations as appropriate (Table 6).

**Table 4:** Dyad-level concordance (N = 218 dyads).

Item	Concordance (%)	κ [95% CI]	Patient+/Family− n(%)	Patient−/Family + n(%)	McNemar p
Independence in ADL	69.3	0.03 [-0.08, 0.15]	49 (73.1%)	18 (26.9%)	< 0.001
Engagement in enjoyable and meaningful activities	67.4	0.10 [-0.04, 0.23]	24 (33.8%)	47 (66.2%)	0.009
Freedom from pain and suffering	67.4	0.14 [0.01, 0.28]	23 (32.4%)	48 (67.6%)	0.004
Being treated with dignity and respect	56	0.15 [0.04, 0.27]	23 (24.0%)	73 (76.0%)	< 0.001
Fulfillment of social roles	58.3	0.17 [0.04, 0.29]	52 (57.1%)	39 (42.9%)	0.208
Financial stability	58.5	0.17 [0.04, 0.32]	39 (43.3%)	51 (56.7%)	0.246
Not being a burden to family	63.3	0.17 [0.03, 0.31]	54 (67.5%)	26 (32.5%)	0.003
Prioritizing palliation over prolonging life	70.2	0.18 [0.04, 0.32]	26 (40.0%)	39 (60.0%)	0.137
Maintaining a strong appearance to others	70.6	0.18 [0.05, 0.34]	29 (45.3%)	35 (54.7%)	0.532
Trust in one's physician	62.8	0.19 [0.06, 0.32]	40 (49.4%)	41 (50.6%)	1
Longevity/Prolongation of life	58.3	0.20 [0.11, 0.32]	21 (23.1%)	70 (76.9%)	< 0.001
Maintaining preferred lifestyle and daily routines	73.4	0.22 [0.07, 0.35]	20 (34.5%)	38 (65.5%)	0.026
Spending sufficient time with family and friends	70.2	0.22 [0.11, 0.37]	22 (33.8%)	43 (66.2%)	0.013
Prognostic awareness	68.3	0.24 [0.10, 0.36]	47 (68.1%)	22 (31.9%)	0.004
Receiving treatment until personal satisfaction is met	60.1	0.26 [0.16, 0.36]	10 (11.5%)	77 (88.5%)	< 0.001
Receiving all possible medical treatments	63.8	0.28 [0.14, 0.41]	26 (32.9%)	53 (67.1%)	0.003
Not being a burden to others	70.6	0.28 [0.15, 0.41]	40 (62.5%)	24 (37.5%)	0.061
Communicating important matters to loved ones	64.7	0.30 [0.17, 0.43]	31 (40.3%)	46 (59.7%)	0.111
Willingness to receive treatment in a facility	67.4	0.31 [0.17, 0.43]	40 (56.3%)	31 (43.7%)	0.342
Support from religious or spiritual beliefs	89.4	0.32 [0.10, 0.51]	8 (34.8%)	15 (65.2%)	0.211

**Table 5:** Discordance by ACP discussion status.

Item	Discordance % (Discussed, n = 122)	Discordance % (Not discussed, n = 96)	p	V
Engagement in enjoyable and meaningful activities	30.3	35.4	0.426	0.05
Fulfillment of social roles	38.5	45.8	0.277	0.07
Spending sufficient time with family and friends	27.9	32.3	0.479	0.05
Independence in ADL	33.6	27.1	0.300	0.07
Longevity/Prolongation of life	38.5	45.8	0.277	0.07
Support from religious or spiritual beliefs	9.8	11.5	0.699	0.03
Financial stability	36.4	47.9	0.086	0.12
Receiving all possible medical treatments	29.5	44.8	0.020	0.16
Being treated with dignity and respect	38.5	51.0	0.065	0.13
Not being a burden to others	27.9	31.2	0.586	0.04
Not being a burden to family	34.4	39.6	0.433	0.05
Maintaining a strong appearance to others	28.7	30.2	0.807	0.02
Communicating important matters to loved ones	32.8	38.5	0.378	0.06
Freedom from pain and suffering	24.6	42.7	0.005	0.19
Prognostic awareness	30.3	33.3	0.636	0.03
Receiving treatment until personal satisfaction is met	39.3	40.6	0.848	0.01
Prioritizing palliation over prolonging life	23.0	38.5	0.012	0.17
Maintaining preferred lifestyle and daily routines	27.0	26.0	0.867	0.01
Trust in one's physician	33.6	41.7	0.221	0.08
Willingness to receive treatment in a facility	32.0	33.3	0.831	0.01
Total discordance score (Mann-Whitney U)	6.0 [4.0-8.0]	7.0 [5.8-9.0]	< 0.001	—

**Table 6:** Associations between total discordance score and clinical/contextual factors.

Part A. Group comparisons (Mann-Whitney *U* test)					
Variable	Group 1	Median [IQR]	Group 2	Median [IQR]	p
*Disease stage*	Early (n = 179)	6.0 [5.0–8.0]	Advanced (n = 32)	8.0 [5.8–9.0]	0.111
*Chemotherapy*	No (n = 111)	6.0 [4.5–8.0]	Yes (n = 107)	7.0 [5.0–9.0]	0.012
*Hormone therapy*	No (n = 59)	7.0 [5.0–9.0]	Yes (n = 158)	6.0 [5.0–8.0]	0.333
*ACP discussion*	Discussed (n = 122)	6.0 [4.0–8.0]	Not discussed (n = 96)	7.0 [5.8–9.0]	< 0.001
*ACP timing*	Pre-surgery (n = 9)	5.0 [3.0–7.0]	Post-surgery (n = 183)	6.0 [5.0–8.5]	0.109

**Part B. Correlations (Spearman rank)**					
**Variable**	**N**	**ρ**	**p**		

*Days from surgery to ACP*	192	−0.02	0.755		
*Patient age*	218	−0.10	0.135		

Multiple correspondence analysis (MCA) was used to derive value and preference dimensions, with respondent category as supplementary; KR-20 coefficients summarized exploratory reliability (Fig. 1A, Fig. 1BA and B; Supplementary Table 3–5). The actor–partner interdependence model (APIM) [19] was then applied to MCA-derived scores: patient models used averaged family value scores as partner variables, while family models retained all respondents with a patient-level random intercept to accommodate the one-to-many structure (94 of 218 patients had ≥2 family respondents). Exploratory APIM extensions examined subgroups defined by surrogate type and dyad concordance level (Supplementary Fig. 1–5; Supplementary Table 6–8).

**Fig. 1A:**
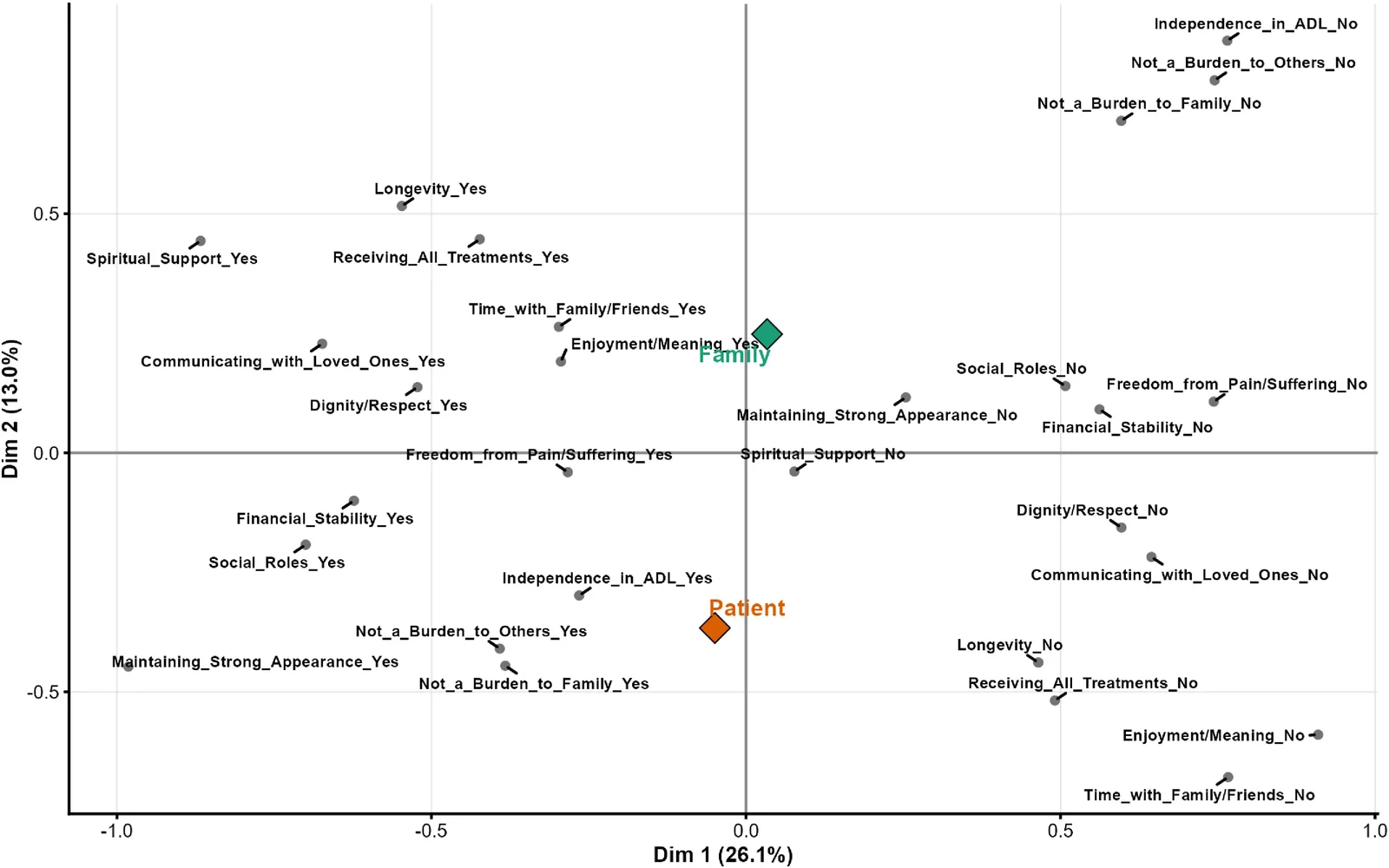
Multiple correspondence analysis of Life Priorities and Values: overall value engagement (Dimension 1) and burden-avoidance versus treatment-seeking (Dimension 2). Biplot of the 14 binary Life Priorities and Values items, based on the pooled responses of 217 breast cancer patients and 322 family respondents drawn from 218 patient–family units at a single Japanese breast cancer unit (August 2024–May 2025). Patients answered for themselves; family members answered what they believed the patient considered important. Plotted points are item response categories. Respondent category (patient versus family) was included as a supplementary variable, meaning that it did not contribute to constructing the dimensions and is shown only as a centroid. Dimension 1 (horizontal, 26.1% of variance) is an overall value-engagement axis that orders respondents by how many life priorities they endorsed (r = −0.99 with the number of items selected): lower (left) scores indicate broad endorsement of many priorities, higher (right) scores a narrow and selective pattern. Dimension 2 (vertical, 13.0%) is a bipolar contrast: higher scores load on “Longevity,” “Receiving all possible medical treatments,” and “Support from religious or spiritual beliefs” (treatment-seeking), and lower scores on “Not being a burden to family,” “Not being a burden to others,” and “Maintaining a strong appearance to others” (burden-avoidance). Items lying close together were endorsed by similar respondents, and distance from the origin indicates how strongly an item discriminates between respondents. The patient and family centroids separate mainly along Dimension 2, with patients positioned toward burden-avoidance and self-reliance and families toward treatment-seeking. ADL, activities of daily living.

**Fig. 1B:**
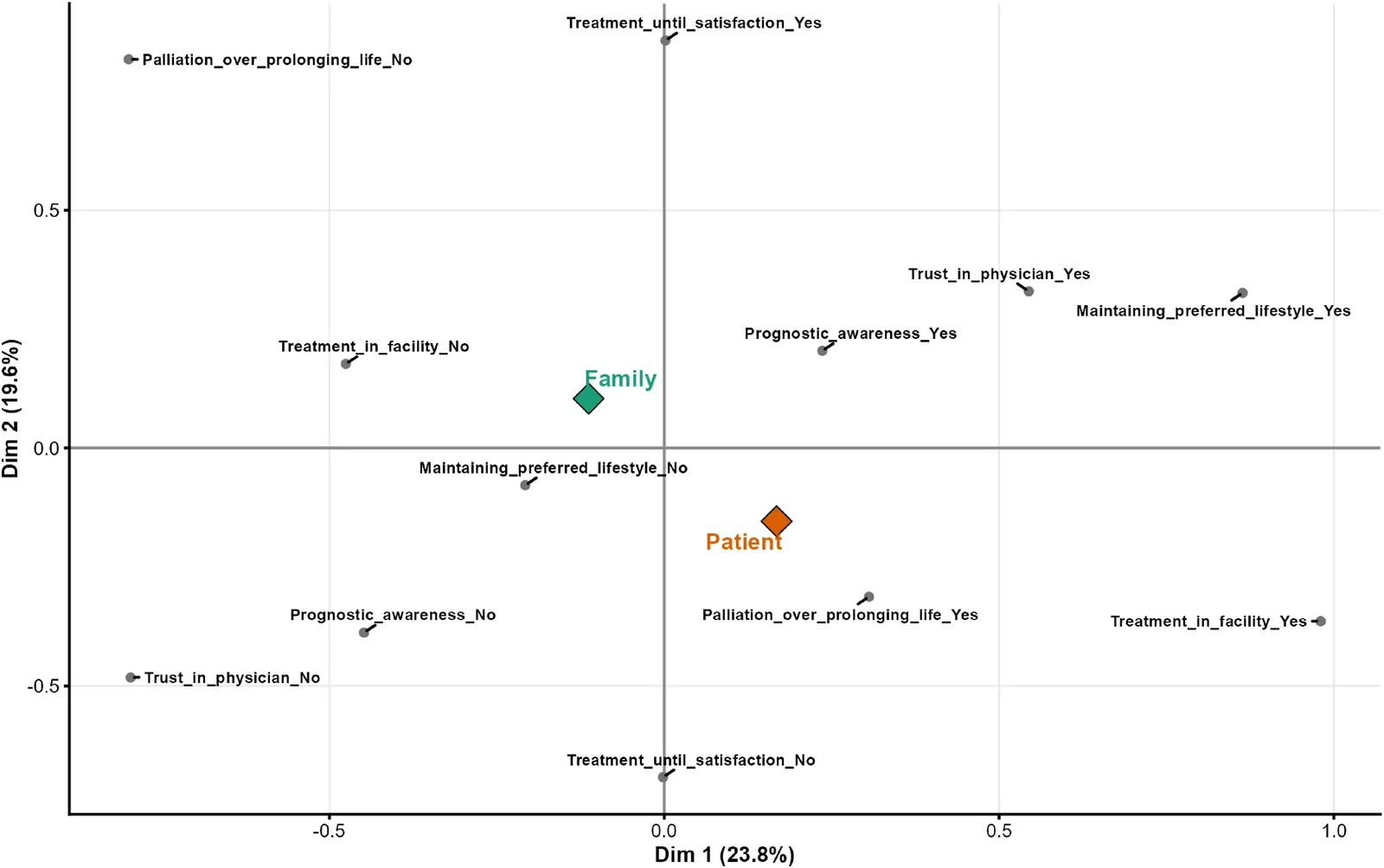
Multiple correspondence analysis of End-of-Life Care Preferences: overall treatment activism (Dimension 1) and active treatment pursuit versus palliative orientation (Dimension 2). Biplot of the 6 analyzed binary End-of-Life Care Preferences items, based on the same pooled sample of 217 patients and 322 family respondents from 218 patient–family units. Patients reported the care they would want for themselves; family members reported the care they would want the patient to receive if the patient became severely ill. Respondent category was again supplementary. Dimension 1 (horizontal, 23.8% of variance) is largely a breadth-of-endorsement axis, but with the opposite sign to Fig. 1A: higher scores correspond to endorsing more preference items (r = +0.92 with the number selected) and therefore to greater overall treatment activism. Dimension 2 (vertical, 19.6%) separates active treatment pursuit at higher scores, loading on “Receiving treatment until personal satisfaction is met,” “Trust in one's physician,” and “Maintaining preferred lifestyle and daily routines,” from a palliative orientation at lower scores, loading on “Prioritizing palliation over prolonging life,” “Prognostic awareness,” and willingness to receive treatment in a facility. Because the two dimensions run in opposite directions with respect to endorsement, the sign of a coefficient linking them should be read against the direction stated here rather than assumed. ADL, activities of daily living.

**Fig. 2:**
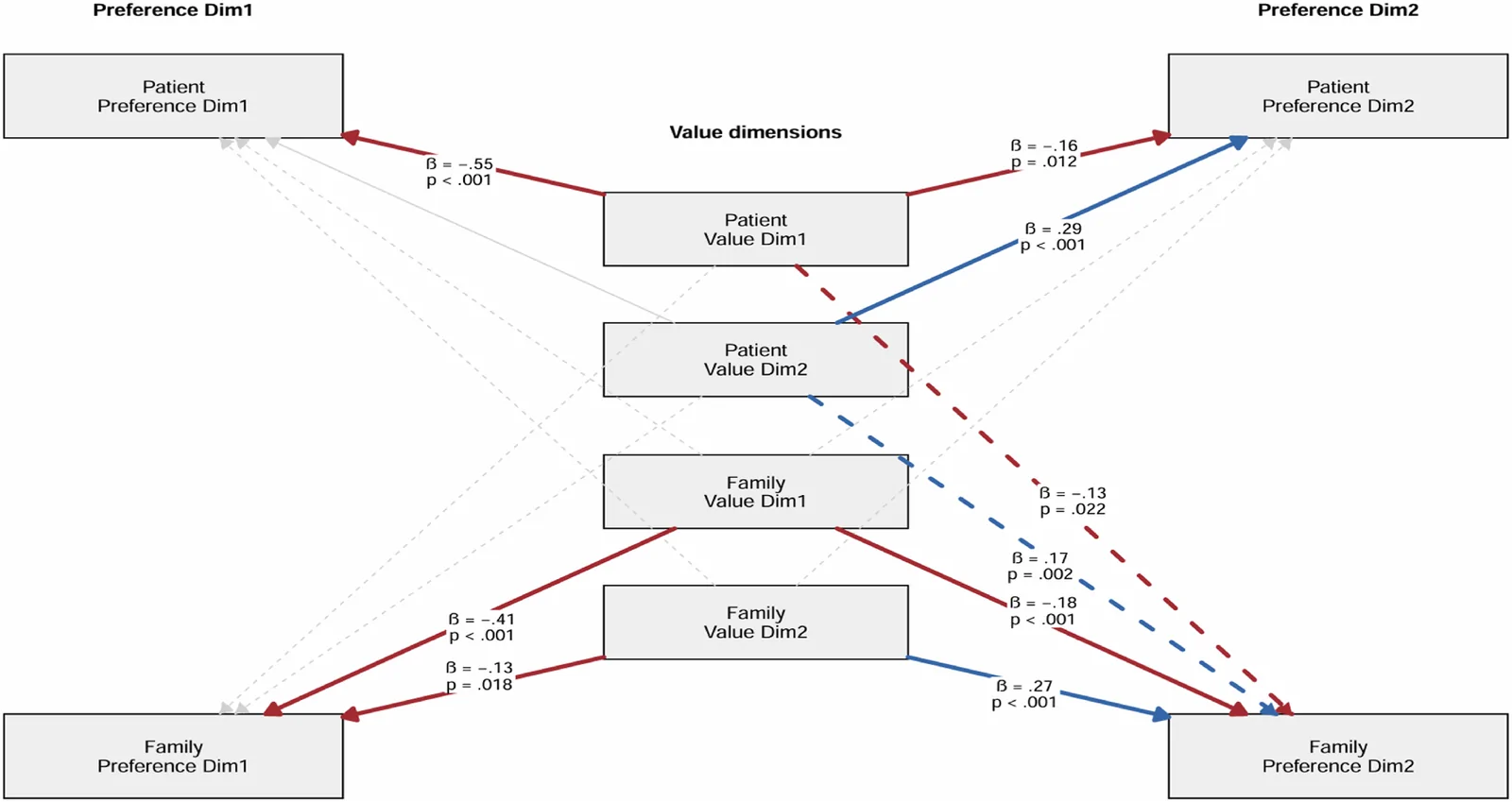
Unified actor–partner interdependence model: how each member's value orientation relates to their own and their partner's treatment preferences. * * Outcomes are labelled by conceptual meaning, with the technical dimension name in parentheses: overall treatment activism (Preference Dimension 1) on the left, and active treatment pursuit versus palliative orientation (Preference Dimension 2) on the right. Predictors, shown in the centre, are overall value engagement (Value Dimension 1) and treatment-seeking versus burden-avoidance (Value Dimension 2); both outcomes are modelled simultaneously. Actor paths run within-role (patient values → patient preferences; family values → family preferences) and partner paths run cross-role. Solid lines with β coefficients denote significant paths (p < .05); dashed grey lines denote non-significant paths. The direction of each axis differs and should be read explicitly: lower Value Dimension 1 scores and higher Preference Dimension 1 scores both denote broader endorsement, so a negative β between them denotes a positive conceptual association between greater value engagement and greater treatment activism. Higher Value Dimension 2 and higher Preference Dimension 2 scores denote a stronger longevity/treatment orientation and stronger active treatment pursuit, respectively. The patient-side model (n = 217) used the mean of each patient's family respondents' value scores as the partner variable; the family-side model (n = 322 responses) retained all family responses with a patient-level random intercept.

**Fig. 3:**
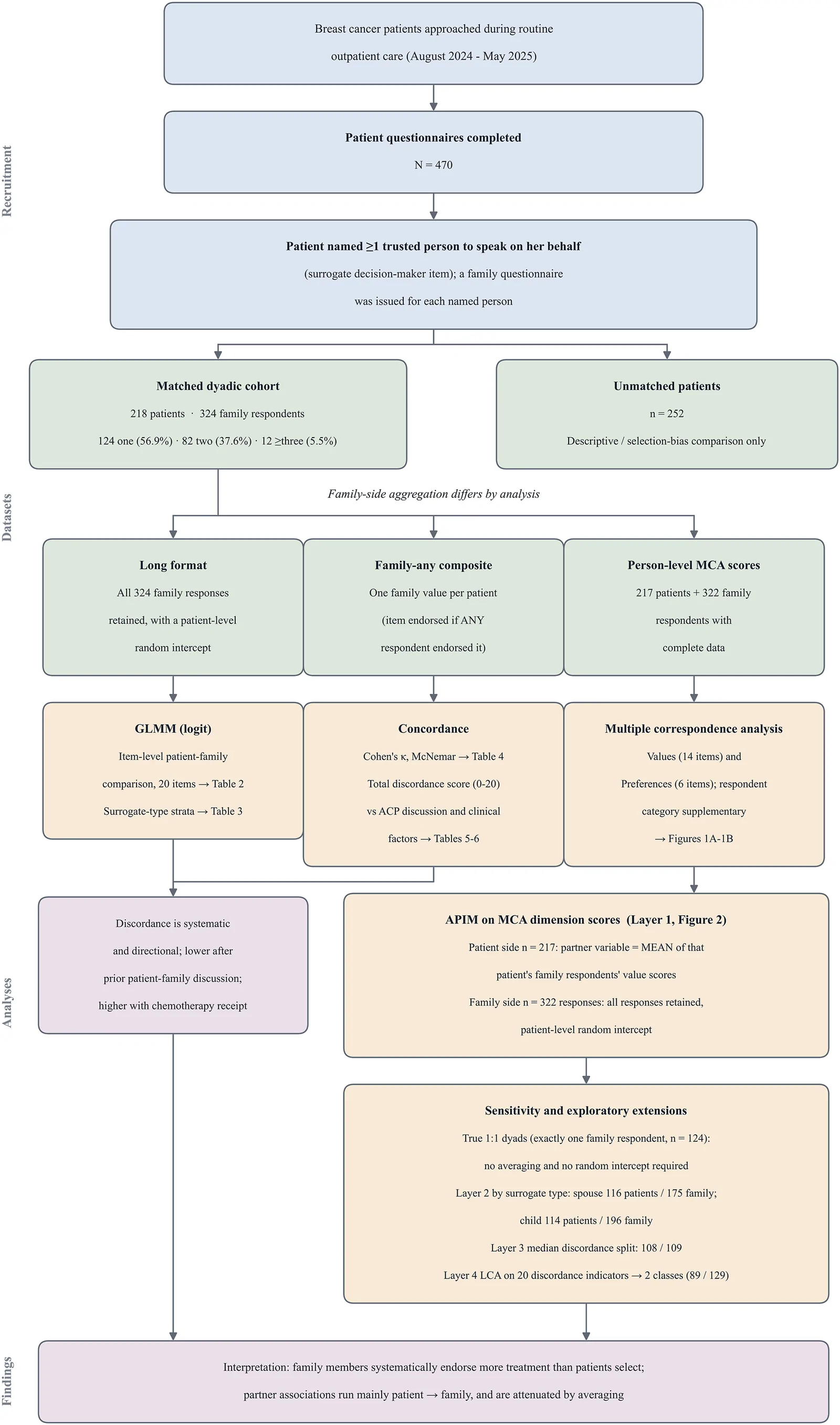
Analytical workflow and the dataset used for each analysis. Flow of participants from recruitment through each analysis, showing which family-side dataset or aggregation was used at each step. Of 470 patients who completed a questionnaire, 218 named at least one surrogate decision-maker who returned a family questionnaire, yielding 324 family respondents; the remaining 252 patients contributed to descriptive and selection-bias comparisons only. Because most patients had more than one family respondent, the family side was handled in three different ways depending on the analysis: the long-format dataset retained all 324 family responses with a patient-level random intercept (GLMM, Table 2, Table 3); the family-any composite reduced each patient to a single family value, an item counting as family-endorsed if any respondent endorsed it (concordance and discordance analyses, Table 4, Table 5, Table 6); and person-level MCA scores were computed for the 217 patients and 322 family respondents with complete data (Fig. 1A, Fig. 1BA and B) and carried forward into the APIM. In APIM Layer 1 (Fig. 2) the patient-side model used the mean of that patient's family respondents' value scores as the partner variable, whereas the family-side model retained all 322 responses with a patient-level random intercept; a sensitivity analysis refitted the model in the 124 true one-to-one dyads. GLMM, generalized linear mixed-effects model; MCA, multiple correspondence analysis; APIM, actor–partner interdependence model; LCA, latent class analysis; ACP, advance care planning.

Fig. 3 summarizes the analytical workflow and identifies the dataset used at each step. Because the design was one-to-many rather than strictly dyadic, different analyses required different family-side aggregations. Item-level patient–family comparisons (GLMM; Table 2) and the surrogate-type subgroup models (Table 3) used the full long-format dataset of 218 patients and all 324 family responses, with a patient-level random intercept accounting for repeated family responses within a patient. Dyad-level concordance analyses (κ, McNemar; Table 4) and the total discordance score (Table 5, Table 6) require a single family value per patient and therefore used a family-any composite, in which an item counted as family-endorsed if any family respondent for that patient endorsed it, yielding 218 dyads each contributing one composite. MCA was fitted to the pooled person-level responses of all patients and family respondents, with respondent category included as a supplementary variable, and produced person-level dimension scores (Fig. 1A, Fig. 1BA and B). APIM Layer 1 (Fig. 2) used those scores: the patient-side model included the 217 patients with complete scores and represented the partner variable as the mean of that patient's family respondents' value scores, whereas the family-side model retained all 322 family responses with a patient-level random intercept. As a sensitivity analysis, Layer 1 was refitted in the 124 patients who had exactly one family respondent, giving true one-to-one dyads that require neither averaging nor a random intercept. Layer 2 subgroup models were restricted by patient-designated surrogate type (spouse-designated: 116 patients, 175 family responses; child-designated: 114 patients, 196 family responses) and retained the same one-to-many structure. Layer 3 split dyads at the median total discordance score, and Layer 4 fitted a latent class analysis to the 20 item-level discordance indicators for the 218 dyads, followed by class-stratified APIM.

### 2.5 Ethics statements

The study was approved by the Institutional Review Board of Jyoban Hospital of Tokiwa Foundation (JHTF-2024-016).

## 3 Results

Participant characteristics are shown in Table 1. The full patient cohort (N = 470) had mean age 62.4 ± 13.7 years, was predominantly post-menopausal (64.9%), and mostly had early-stage disease (stage I–II: 78.7%). The matched dyadic sample included 218 patients (mean age 64.2 ± 13.2) and 324 family members (mean age 52.5 ± 15.5). Among the 218 matched patients, 124 (56.9%) had one family respondent, 82 (37.6%) had two, and 12 (5.5%) had three or more; these percentages use matched patients as the denominator and count every returned family questionnaire, not only the questionnaire from the person the patient named first. Among the 324 family respondents, 157 (48.5%) reported having previously talked with the patient about end-of-life topics, 125 (38.6%) had not, and 8 (2.5%) were unwilling to do so; the remaining 34 (10.4%) did not answer this item. Spouses (53.2%) and children (52.3%) were the most frequently designated surrogate decision-makers; these proportions sum to more than 100% because patients could name more than one person. Matched and unmatched patients showed no clinically meaningful differences across ACP items or surrogate designations (Cramér's V < 0.12; Supplementary Table 1).

GLMMs showed systematic patient-family divergence (Table 2). Patients more often endorsed autonomy-related priorities: family members were less likely than patients to endorse "Independence in ADL" (OR = 0.20, 95% CI [0.12, 0.34], p < .001), "Not being a burden to family" (OR = 0.31 [0.20, 0.48], p < .001), "Not being a burden to others" (OR = 0.37 [0.24, 0.59], p < .001), and "Prognostic awareness" (OR = 0.32 [0.20, 0.51], p < .001). Families more often endorsed intervention-oriented priorities, including "Longevity" (OR = 2.15 [1.43, 3.24], p < .001), "Being treated with dignity" (OR = 1.90 [1.31, 2.76], p < .001), and most prominently "Receiving treatment until satisfaction" (OR = 4.50 [2.75, 7.37], p < .001, ICC = 0.40).

Surrogate-type stratification (Table 3; Supplementary Table 2) showed that broad autonomy and treatment-intensity gaps persisted across subgroups, with relationship-specific differences. When a spouse was designated, "Treatment until satisfaction" showed a larger OR (7.18 [3.35, 15.37], p < .001) than the overall estimate, indicating particularly large spouse-associated divergence on aggressive treatment. "Willingness to receive treatment in a facility" was significant only in spouse-designated dyads (OR = 0.35 [0.18, 0.67], Bonferroni-adjusted p = .003). Child-designated dyads showed a specific difference for "Trust in physician" (OR = 0.51 [0.30, 0.89], Bonferroni-adjusted p = .034).

Concordance was poor and directional (Table 4). Cohen's κ ranged from 0.03 to 0.32 across the 20 items, and the mean total discordance score was 6.7 ± 3.1, meaning that the average dyad disagreed on approximately one-third of items. For "Receiving treatment until satisfaction" (κ = 0.26), 88.5% of discordant pairs were Family+/Patient− (McNemar p < .001); for "Longevity," 76.9% were Family+/Patient− (p < .001). In contrast, "Independence in ADL" (κ = 0.03) showed 73.1% Patient+/Family− discordance (p < .001), and "Not being a burden to family" showed 67.5% in the same direction (p = .003).

Prior ACP discussion was associated with lower total discordance (median 6 [IQR 4–8] vs. 7 [5.8–9.0], p < .001; Table 5). This association was most evident for palliative care items: discordance on "Freedom from pain" was 24.6% in the Discussed group versus 42.7% in the Not Discussed group (p = .005, V = 0.19), "Palliation over prolongation" was 23.0% versus 38.5% (p = .012, V = 0.17), and "Receiving all treatments" was 29.5% versus 44.8% (p = .020, V = 0.16). Among clinical factors, chemotherapy or targeted therapy was associated with higher total discordance (median 7 vs. 6, p = .012; Table 6). Discordance was numerically higher in the advanced-stage subgroup, but the difference was not statistically significant (median 8 vs. 6, p = .111; n = 32); patient age, time since surgery, hormone therapy, and ACP timing were likewise not significantly associated with discordance.

MCA identified two Life Priorities dimensions (Fig. 1A). Dimension 1 (26.1% of variance) was an overall value-engagement axis: it ordered respondents almost perfectly by the number of value items endorsed (r = −0.99 between the dimension score and the count of items selected), so that lower scores denote broad endorsement of many life priorities and higher scores denote a narrow, selective response pattern. Dimension 2 (13.0%) was a bipolar contrast between burden-avoidance and treatment-seeking: higher scores loaded on “Longevity,” “Receiving all possible medical treatments,” and “Support from religious or spiritual beliefs,” and lower scores on “Not being a burden to family,” “Not being a burden to others,” and “Maintaining a strong appearance to others.” For End-of-Life Care Preferences (Fig. 1B), Dimension 1 (23.8%) was likewise largely a breadth-of-endorsement axis but with the opposite sign (r = +0.92 with the number of preference items endorsed), so that higher scores denote greater overall treatment activism. Dimension 2 (19.6%) separated active treatment pursuit at higher scores, loading on “Receiving treatment until personal satisfaction is met,” “Trust in one's physician,” and “Maintaining preferred lifestyle and daily routines,” from a palliative orientation at lower scores, loading on “Prioritizing palliation over prolonging life,” “Prognostic awareness,” and willingness to receive treatment in a facility. Patient and family centroids diverged mainly along the treatment-seeking axes, with patients positioned toward self-reliance and families toward dependency for survival. KR-20 coefficients for the Life Priorities dimensions were 0.74 and 0.53 (Supplementary Table 5; MCA coordinates in Supplementary Table 3 and Supplementary Table 4).

APIM using MCA scores (Fig. 2) showed strong actor associations. Because the sign of an MCA dimension is arbitrary, the direction of each dimension is stated explicitly here: lower Value Dim1 scores indicate greater endorsement of life priorities, whereas higher Preference Dim1 scores indicate greater endorsement of treatment preferences, and higher Value Dim2 and higher Preference Dim2 scores indicate a stronger longevity/treatment orientation and stronger active treatment pursuit, respectively. A negative β linking Value Dim1 to a preference outcome therefore denotes a positive conceptual association, that is, greater value engagement together with greater treatment orientation. In clinical terms, respondents who endorsed a wider range of life priorities also endorsed a wider range of active treatment preferences. For patients, greater value engagement was associated with higher treatment activism (Preference Dim1: β = −0.55, p < .001) and with active treatment pursuit (Preference Dim2: β = −0.16, p = .012); a stronger longevity/treatment orientation was associated with active treatment pursuit (β = 0.29, p < .001). For family members, predicting greater patient value engagement was associated with higher treatment activism (β = −0.41, p < .001) and active treatment pursuit (β = −0.18, p < .001), and predicting a stronger longevity/treatment orientation was associated with active treatment pursuit (β = 0.27, p < .001). Patient–family value correlations were modest (Value Dim1: r = 0.18, p = .009; Value Dim2: r = 0.29, p < .001), indicating partial but imperfect alignment.

No patient-side partner association reached significance (all p > .23). Two family-side partner associations were observed for Preference Dim2: patients' greater value engagement was associated with family active treatment pursuit (β = −0.13, p = .022), and patients' stronger longevity/treatment orientation showed the same pattern (β = 0.17, p = .002). Because the patient-side model averaged family value scores whereas the family-side model retained individual responses, the Layer 1 model was repeated in the 124 patients with exactly one family respondent, that is, true one-to-one dyads requiring neither averaging nor a random intercept. Actor associations were essentially unchanged (patient Preference Dim1 on patient Value Dim1: β = −0.55, p < .001; family Preference Dim1 on family Value Dim1: β = −0.41, p < .001). The partner association from patients' longevity/treatment orientation to family active treatment pursuit was in the same direction and larger than in the averaged model (β = 0.28, p = .001 versus β = 0.17, p = .002), and the partner association from patients’ value engagement was in the same direction but no longer significant in the smaller sample (β = −0.11, p = .21 versus β = −0.13, p = .022). No patient-side partner association was significant in either model. Exploratory extensions suggested contextual heterogeneity: spouse dyads (n = 116) showed more bidirectional partner patterns, child dyads (n = 114) mainly patient-to-family associations, and a 2-class LCA solution identified generally concordant (n = 89, 40.8%) and value-discordant classes (n = 129, 59.2%; BIC = 5542, entropy = 0.626). The value-discordant class had lower prior ACP discussion (48.8% vs. 66.3%, p = .016) and higher chemotherapy prevalence (55.0% vs. 40.4%, p = .048) (Supplementary Fig. 1–5; Supplementary Table 6–8).

## 4 Discussion

This study demonstrates systematic, directional patient-family discordance in end-of-life values and treatment preferences among Japanese breast cancer dyads. Patients emphasized autonomy, independence, and non-burden, whereas family members' predictions and treatment preferences more often favored survival and active treatment. This pattern is consistent with previous studies showing family overestimation of life-prolonging preferences, [9,10] but the present analysis extends that literature by quantifying the direction of disagreement item by item. The finding that 88.5% of discordance on treatment pursuit was concentrated on the family-endorsement side is clinically important: when conflict emerges around treatment intensity, the more common pattern may be family preference for more treatment than the patient selected, rather than random disagreement.

This framing also changes how discordance should be interpreted. The analyses do not contrast two independent sets of personal values; they compare patient-reported priorities with family predictions and family treatment intentions on the patient's behalf. Accordingly, low κ values represent limited shared understanding of the patient's wishes as much as preference disagreement. For ACP practice, improving concordance requires more than recording a surrogate's identity; it requires testing whether the surrogate's assumptions match the patient's stated priorities.

Prior ACP discussion was associated with lower discordance, especially for palliative items. Because this was a cross-sectional study, the direction of the association cannot be inferred; dyads with more aligned values may have been more likely to discuss ACP. Still, the concentration of the association in “Freedom from pain,” “Palliation over prolongation,” and “Receiving all treatments” suggests that discussion may be particularly relevant when decisions involve shifting from treatment pursuit toward comfort-oriented goals. Chemotherapy receipt was also associated with higher discordance (p = .012). Because the data are cross-sectional and treatment was not randomized, this association cannot be interpreted causally, and several non-causal explanations are plausible. Patients receiving chemotherapy face more complex, multi-step treatment decisions, so there is simply more about which a dyad can disagree. They may also have more advanced or biologically less favourable disease, so that families confront a poorer prognosis and may respond by favouring more aggressive care. Chemotherapy imposes a greater practical and emotional caregiving burden on families, which may itself shift what families want for the patient. Communication within the dyad also changes over the disease course, so the association may partly reflect where a dyad stands in its illness trajectory rather than any effect of treatment itself. Reverse or shared causation is likewise possible, in that dyads with divergent values may make different treatment choices in the first place. Whatever the mechanism, treatment transitions appear to be useful moments at which to reassess whether patients and family members understand each other's priorities.

The MCA-APIM analysis adds a dimension-level view of dyadic interdependence. Actor associations supported the coherence of the MCA-derived dimensions: patients' own value orientations were related to their own treatment preferences, and families' predictions of patient value orientations were related to what families wanted the patient to receive. Partner associations were more asymmetric. Patients' value engagement and longevity/treatment orientation were associated with families' active treatment pursuit for the patient, whereas family-derived value dimensions were not significantly associated with patient preference outcomes. The asymmetry of the two models also warrants comment. Because most patients contributed more than one family respondent, a strictly dyadic APIM was not estimable without discarding data. The partner variable in the patient-side model was therefore represented as the mean of that patient's family respondents' value scores, which is the conventional approach when the partner is a set of individuals rather than one person and which retains all 217 patients; the family-side model instead retained every family response with a patient-level random intercept, because there the repeated observations constitute the outcome and multilevel modelling is the appropriate treatment. The two sides are consequently not symmetric in how they handle within-family variability. Averaging removes between-respondent variance on the patient side and biases partner estimates toward the null, whereas the family side preserves that variance. Actor and partner estimates from the two sides are therefore not directly comparable in magnitude, and the absence of significant patient-side partner effects should be read as weak evidence rather than as evidence of absence. The sensitivity analysis restricted to true one-to-one dyads supports this reading: the partner association from patients' longevity orientation to family active treatment pursuit was larger, not smaller, when no averaging was applied. This pattern suggests that family treatment expectations may be more closely linked to patient-reported priorities than patient preferences are to family predictions, although shared context, disease experience, and communication patterns may also contribute. Subgroup and LCA findings further suggest that relationship type and overall concordance level may shape these associations, but these exploratory results require replication.

The results have practical implications for ACP in outpatient breast cancer care. Existing guidance already recommends that ACP be initiated early and revisited at clinical milestones: ASCO recommends integrating palliative care and goals-of-care discussion from the point of advanced diagnosis, ESMO makes comparable recommendations for supportive and palliative care, and international consensus definitions of ACP emphasize iterative discussion involving both patients and those who may later speak for them [[20], [21], [22]]. Our findings are largely confirmatory of that guidance rather than contradictory of it, but they add two elements that milestone-based recommendations do not by themselves supply. First, they quantify how far surrogate understanding actually diverges from patient priorities in a routine, largely early-stage oncology population, and show that the divergence is directional rather than random. Second, they identify the content on which such discussions most need to focus: the largest gaps concerned treatment intensity and burden-related priorities rather than communication in general. Clinicians should therefore not assume that family members accurately predict patients’ priorities, particularly when spouses are designated surrogates and aggressive treatment is under discussion.

These findings also connect to a broader literature on dyadic and surrogate decision-making. Surrogates frequently report feeling unprepared for the role and describe wanting more explicit prior conversation with the patient [17,18], and structured communication frameworks such as the Serious Illness Conversation Guide have been developed to make values elicitation systematic rather than ad hoc [23]. Our data suggest where such a framework would add most value in this setting. Because family members were on average systematically biased toward more treatment than patients selected, simply asking a surrogate what the patient would want is unlikely to be sufficient. A brief structured instrument completed independently by both members of the dyad, followed by explicit comparison of the two sets of answers, would make the direction and the content of any mismatch visible to the clinician rather than leaving it to be discovered during a crisis. For spouse dyads, discussion may need to focus on expectations about treatment intensity; for parent–child dyads, shared trust in the care team may deserve particular attention [17,18]. At chemotherapy initiation, at completion, and around recurrence-related transitions, revisiting ACP may help identify widening patient–family value gaps before urgent decisions arise.

These patterns should be read against the specific structure of family decision-making in Japan, which differs in several respects from the individual-autonomy model underpinning much Western ACP guidance. Disclosure and treatment decisions in Japan have historically been family-mediated rather than patient-centred, and it remains common for clinicians to speak with the family alongside, and occasionally before, the patient [11,12]. Responsibility within the family is also distributed by position rather than shared equally: the household head, most often the husband in older cohorts, and the eldest child commonly assume the representative role, which helps explain why spouses and children accounted for the large majority of designated surrogates in this cohort and why spouse-designated dyads showed the largest divergence on aggressive treatment. Age and gender compound this pattern. Patients here were on average about 12 years older than their family respondents, and a generational gap in expectations about disclosure and life-prolonging treatment is well described in Japan; older patients are also more likely to frame their own priorities in terms of not burdening the family, which is precisely the domain in which family members most underestimated them. Norms of restraint in voicing personal wishes within the family, such that a preference for comfort-oriented care may go unstated so as not to appear to be giving up or to burden others, offer a plausible account of the directional bias observed here, in which families endorsed more treatment than patients selected in 88.5% of discordant pairs on treatment pursuit. For international readers, the practical implication is that the low concordance reported here should not be read simply as poor communication. It reflects a setting in which the surrogate role is culturally assumed rather than explicitly negotiated, which is what makes structured and explicit dyadic elicitation valuable.

## 5 Limitations

Several limitations should be noted. First, this was a single-center cross-sectional study of breast cancer patients, limiting generalizability and precluding causal inference. Second, questionnaires were physician-administered and non-anonymous, which may have introduced social desirability bias. Third, treatment preferences may change over time, particularly with disease progression, and this design cannot capture longitudinal shifts. Fourth, convenience sampling without documented refusal rates limits inference about the broader patient population. Fifth, family background data, including illness understanding, prior caregiving experience, and consultation attendance, were limited; the relationship of each family respondent to the patient was recorded only as free text and was categorized post hoc. Sixth, although the item pool was informed by established ACP frameworks and refined through clinical consensus [1,13,14], the questionnaire was developed internally for this study and has not undergone formal psychometric validation: an early feasibility review of item usability and response distributions was carried out during data collection, but no cognitive interviewing, reliability testing, or confirmatory factor-analytic work was performed, and the KR-20 coefficients reported here are exploratory and were derived from the same sample in which the dimensions were estimated. The instrument should therefore be regarded as a content-valid clinical tool rather than a validated measurement scale, and item-level estimates may not transfer unchanged to other settings. Seventh, the cohort was predominantly early-stage: 78.7% of the full cohort had stage I–II disease and only 5.7% had stage IV disease (8.7% among matched patients), so these findings describe ACP in a largely early breast cancer population rather than in metastatic disease. Whether discordance is as large in metastatic breast cancer cannot be resolved here. It might plausibly be lower, because a poorer and more explicitly communicated prognosis, more frequent goals-of-care discussion, and longer shared treatment experience may align expectations; it might equally be higher if families intensify their preference for life-prolonging treatment as options narrow. The advanced-stage subgroup in this study was too small for a stable estimate (n = 32, p = .111). A dedicated study restricted to metastatic breast cancer, ideally longitudinal with repeated measurement across lines of therapy, is the appropriate next step. Relatedly, family members in this cohort had often already accompanied the patient through prior treatment, and that shared experience may itself shape what they would choose for her: families who have witnessed toxicity or benefit at first hand may diverge from the patient for reasons unrelated to any misunderstanding of her values, which is an additional and under-examined source of discordance. Finally, MCA and LCA findings are exploratory and sample-specific; LCA entropy was moderate (0.626), and the APIM structure was one-to-many rather than strictly dyadic, so averaging family scores and using multilevel models may attenuate partner associations relative to matched single-pair designs, as the one-to-one sensitivity analysis indicated.

## 6 Conclusion

Patient-family discordance in end-of-life values and treatment preferences was common, directional, and associated with prior ACP discussion and chemotherapy history. MCA-APIM analyses suggested that family treatment preferences were associated with both family predictions of patient values and patients' own value orientations. Effective ACP should therefore elicit individual preferences, make family predictions explicit, and address dyadic value alignment before treatment decisions become urgent.

## CRediT authorship contribution statement

**Akemi Hara:** Conceptualization, Formal analysis, Investigation, Methodology, Writing – original draft. **Ayu Ajitomi:** Conceptualization, Data curation, Investigation, Methodology, Writing – original draft. **Kazunoshin Tachibana:** Conceptualization, Writing – review & editing. **Kenji Gonda:** Conceptualization, Writing – review & editing. **Masahiro Wada:** Conceptualization, Writing – review & editing. **Rui Omori:** Conceptualization, Writing – review & editing. **Megumi Arai:** Conceptualization, Writing – review & editing. **Kayoko Konuma:** Conceptualization, Writing – review & editing. **Toyoaki Sawano:** Conceptualization, Writing – review & editing. **Yoshiaki Kanemoto:** Conceptualization, Writing – review & editing. **Hirotomo Miyatake:** Conceptualization, Writing – review & editing. **Tomohiro Kurokawa:** Conceptualization, Writing – review & editing. **Yukiko Kouchi:** Conceptualization, Writing – review & editing. **Norio Kanzaki:** Conceptualization, Writing – review & editing. **Yasuhiro Kotera:** Conceptualization, Methodology, Writing – review & editing. **Yoshitake Takebayashi:** Conceptualization, Investigation, Methodology, Writing – review & editing. **Michio Murakami:** Conceptualization, Investigation, Methodology, Supervision, Writing – review & editing. **Tohru Ohtake:** Conceptualization, Writing – review & editing. **Hiroaki Shimmura:** Conceptualization, Writing – review & editing. **Akihiko Ozaki:** Conceptualization, Data curation, Methodology, Project administration, Supervision, Writing – review & editing.

## Funding

This study was supported by a Grant-in-Aid for Scientific Research (26K13544) from the 10.13039/501100001691Japan Society for the Promotion of Science.

## Declaration of competing interests

The authors declare the following financial interests/personal relationships which may be considered as potential competing interests: Akihiko Ozaki reports a relationship with MNES that includes: consulting or advisory. Akihiko Ozaki reports a relationship with Kyowa Kirin Inc that includes: speaking and lecture fees. Akihiko Ozaki reports a relationship with Becton Dickinson and Company that includes: speaking and lecture fees. Akihiko Ozaki reports a relationship with Pfizer that includes: speaking and lecture fees. Akihiko Ozaki reports a relationship with Daiichi Sankyo Inc that includes: speaking and lecture fees. Akihiko Ozaki reports a relationship with Taiho Pharmaceutical Co Ltd that includes: speaking and lecture fees. If there are other authors, they declare that they have no known competing financial interests or personal relationships that could have appeared to influence the work reported in this paper.
