# Supplementary material for: End-of-life care values and treatment preferences among Japanese breast cancer patients and families: A dyadic cross-sectional study
Source: Breast. 2026 Aug 12;89:104899. doi: 10.1016/j.breast.2026.104899 (PMC13521167; doi:10.1016/j.breast.2026.104899)
Supplement: Supplementary file 1 [file mmc1.docx]

**Supplementary Table 1. Comparison of matched vs unmatched patients**

| **Item** | **χ²** | **p** | **V** |
| --- | --- | --- | --- |
| Engagement in enjoyable and meaningful activities | 0.0486052154935111 | .826 | .01 |
| Fulfillment of social roles | 3.55007384017962 | .060 | .09 |
| Spending sufficient time with family and friends | 0.163597005836325 | .686 | .02 |
| Independence in ADL | 1.80385595047267 | .179 | .06 |
| Longevity / Prolongation of life | 0.104960015946096 | .746 | .01 |
| Support from religious or spiritual beliefs | 0.969123877152954 | .325 | .05 |
| Financial stability | 3.13972500940518 | .076 | .08 |
| Receiving all possible medical treatments | 2.28124241385712 | .131 | .07 |
| Being treated with dignity and respect | 0.349926620517151 | .554 | .03 |
| Not being a burden to others | 1.69590677955492 | .193 | .06 |
| Not being a burden to family | 0.966749943392504 | .325 | .05 |
| Maintaining a strong appearance to others | 0.0674359774379194 | .795 | .01 |
| Communicating important matters to loved ones | 1.51078063379131 | .219 | .06 |
| Freedom from pain and suffering | 1.14497066584927 | .285 | .05 |
| Prognostic awareness | 0.272722062763565 | .602 | .02 |
| Receiving treatment until personal satisfaction is met | 0.000166663829835538 | .990 | .00 |
| Prioritizing palliation over prolonging life | 2.50924207135856 | .113 | .07 |
| Maintaining preferred lifestyle and daily routines | 0.599092440647104 | .439 | .04 |
| Trust in one's physician | 3.97844205975684 | .046 | .09 |
| Willingness to receive treatment in a facility | 6.23028754574238 | .013 | .12 |
| Surrogate decision-maker: Mother | 2.19641404993431 | .138 | .07 |
| Surrogate decision-maker: Father | 2.94367343703685 | .086 | .08 |
| Surrogate decision-maker: Siblings | 0.233194562596048 | .629 | .02 |
| Surrogate decision-maker: Spouse | 0.906031997983499 | .341 | .04 |
| Surrogate decision-maker: Children | 3.83047328418274 | .050 | .09 |
| Surrogate decision-maker: Cousin | 0.0827942896602263 | .774 | .01 |
| Surrogate decision-maker: Friend | 4.73904047919829 | .029 | .10 |

**Supplementary Table 2. Surrogate designation and ACP item endorsement (χ² tests)** **†**

|  | **Patient** | | **Family** | |
| --- | --- | --- | --- | --- |
| **Item** | **Spouse** | **Child** | **Spouse** | **Child** |
| **Life Priorities and Values** | | | | |
| Engagement in enjoyable and meaningful activities | .12 | .07 | .13 [.02, .24]* | .01 |
| Fulfillment of social roles | .03 | .08 | .05 | .08 |
| Spending sufficient time with family and friends | .16 [.03, .30]* | .07 | .10 | .02 |
| Independence in ADL | .02 | .05 | .01 | .07 |
| Longevity / Prolongation of life | .04 | .08 | .03 | .04 |
| Support from religious or spiritual beliefs | .00 | .01 | .01 | .01 |
| Financial stability | .14 [.00, .28]* | .04 | .05 | .08 |
| Receiving all possible medical treatments | .06 | .00 | .08 | .08 |
| Being treated with dignity and respect | .01 | .04 | .00 | .06 |
| Not being a burden to others | .09 | .05 | .11 | .01 |
| Not being a burden to family | .04 | .07 | .10 | .02 |
| Maintaining a strong appearance to others | .19 [.06, .32]** | .04 | .03 | .03 |
| Communicating important matters to loved ones | .11 | .04 | .03 | .02 |
| Freedom from pain and suffering | .10 | .05 | .03 | .05 |
| **End-of-Life Care Preferences** | | | | |
| Prognostic awareness | .25 [.12, .39]*** | .00 | .15 [.04, .26]** | .05 |
| Receiving treatment until personal satisfaction is met | .05 | .04 | .15 [.05, .26]** | .05 |
| Prioritizing palliation over prolonging life | .17 [.04, .30]* | .03 | .02 | .08 |
| Maintaining preferred lifestyle and daily routines | .05 | .05 | .08 | .02 |
| Trust in one’s physician | .01 | .03 | .02 | .06 |
| Willingness to receive treatment in a facility | .03 | .10 | .05 | .01 |

*† Cramér’s V from χ² tests; 95% CI shown for significant associations. *p < .05, **p < .01, ***p < .001.*

**Supplementary Table 3. MCA coordinates: Life Priorities and Values**

| **Label** | **English name** | **Dim 1** | **Dim 2** |
| --- | --- | --- | --- |
| v01_No | Enjoyment/Meaning_No | .910 | -.590 |
| v01_Yes | Enjoyment/Meaning_Yes | -.294 | .191 |
| v02_No | Social_Roles_No | .508 | .140 |
| v02_Yes | Social_Roles_Yes | -.700 | -.192 |
| v03_No | Time_with_Family/Friends_No | .767 | -.678 |
| v03_Yes | Time_with_Family/Friends_Yes | -.298 | .263 |
| v04_No | Independence_in_ADL_No | .765 | .862 |
| v04_Yes | Independence_in_ADL_Yes | -.265 | -.299 |
| v05_No | Longevity_No | .465 | -.438 |
| v05_Yes | Longevity_Yes | -.547 | .516 |
| v06_No | Spiritual_Support_No | .077 | -.039 |
| v06_Yes | Spiritual_Support_Yes | -.867 | .443 |
| v07_No | Financial_Stability_No | .562 | .091 |
| v07_Yes | Financial_Stability_Yes | -.623 | -.101 |
| v08_No | Receiving_All_Treatments_No | .491 | -.518 |
| v08_Yes | Receiving_All_Treatments_Yes | -.423 | .447 |
| v09_No | Dignity/Respect_No | .597 | -.157 |
| v09_Yes | Dignity/Respect_Yes | -.522 | .137 |
| v10_No | Not_a_Burden_to_Others_No | .745 | .779 |
| v10_Yes | Not_a_Burden_to_Others_Yes | -.391 | -.410 |
| v11_No | Not_a_Burden_to_Family_No | .597 | .694 |
| v11_Yes | Not_a_Burden_to_Family_Yes | -.383 | -.445 |
| v12_No | Maintaining_Strong_Appearance_No | .254 | .116 |
| v12_Yes | Maintaining_Strong_Appearance_Yes | -.982 | -.447 |
| v13_No | Communicating_with_Loved_Ones_No | .644 | -.218 |
| v13_Yes | Communicating_with_Loved_Ones_Yes | -.674 | .228 |
| v14_No | Freedom_from_Pain/Suffering_No | .744 | .107 |
| v14_Yes | Freedom_from_Pain/Suffering_Yes | -.283 | -.041 |

**Supplementary Table 4. MCA coordinates: End-of-Life Care Preferences**

| **Label** | **English name** | **Dim 1** | **Dim 2** |
| --- | --- | --- | --- |
| m01_No | Prognostic_awareness_No | -.448 | -.388 |
| m01_Yes | Prognostic_awareness_Yes | .236 | .205 |
| m02_No | Treatment_until_satisfaction_No | -.002 | -.691 |
| m02_Yes | Treatment_until_satisfaction_Yes | .002 | .857 |
| m03_No | Palliation_over_prolonging_life_No | -.799 | .818 |
| m03_Yes | Palliation_over_prolonging_life_Yes | .306 | -.313 |
| m04_No | Maintaining_preferred_lifestyle_No | -.207 | -.078 |
| m04_Yes | Maintaining_preferred_lifestyle_Yes | .863 | .326 |
| m05_No | Trust_in_physician_No | -.797 | -.483 |
| m05_Yes | Trust_in_physician_Yes | .544 | .330 |
| m06_No | Treatment_in_facility_No | -.475 | .177 |
| m06_Yes | Treatment_in_facility_Yes | .980 | -.365 |

**Supplementary Table 5. Reliability analysis (KR-20)**

| **Scale** | **Items** | **KR-20** |
| --- | --- | --- |
| Dimension 1 | 10 | 0.74 |
| Dimension 2 | 4 | 0.53 |

**Supplementary Table 6. APIM results for spouse dyads (n = 116 patients, 175 family members)** **†**

|  | **Patient outcomes** | | **Family outcomes** | |
| --- | --- | --- | --- | --- |
|  | **Pref Dim1 (R² = 0.27)** | **Pref Dim2 (R² = 0.15)** | **Pref Dim1 (ICC = 0.17)** | **Pref Dim2** |
| **Actor effects** | | | | |
| Value Dim1 | -0.51*** | -.06 | -0.44*** | -0.20** |
| Value Dim2 | -.06 | 0.25** | -.08 | 0.34*** |
| **Partner effects** | | | | |
| Value Dim1 | -.03 | -0.22* | -.08 | -0.15* |
| Value Dim2 | .02 | .14 | .00 | 0.22** |

*† Standardized β from APIM. Actor = own values → own preferences; Partner = other member's values → own preferences. *p < .05, **p < .01, ***p < .001.*

**Supplementary Table 7. APIM results for child dyads (n = 114 patients, 196 family members)** **†**

|  | **Patient outcomes** | | **Family outcomes** | |
| --- | --- | --- | --- | --- |
|  | **Pref Dim1 (R² = 0.37)** | **Pref Dim2 (R² = 0.16)** | **Pref Dim1 (ICC = 0.20)** | **Pref Dim2 (ICC = 0.33)** |
| **Actor effects** | | | | |
| Value Dim1 | -0.58*** | -.12 | -0.46*** | -0.22*** |
| Value Dim2 | .14 | 0.25** | -.13 | 0.23*** |
| **Partner effects** | | | | |
| Value Dim1 | -.03 | -.04 | -.08 | -.13 |
| Value Dim2 | -.10 | .18 | .12 | 0.19* |

*† Standardized β from APIM. Actor = own values → own preferences; Partner = other member's values → own preferences. *p < .05, **p < .01, ***p < .001.*

**Supplementary Table 8A. Latent class model selection**

| **Classes** | **Log-likelihood** | **AIC** | **BIC** | **Entropy** | **Class sizes** | **Selected** |
| --- | --- | --- | --- | --- | --- | --- |
| 1 | -2724.62 | 5489.25 | 5556.94 | — | 218 |  |
| 2 | -2660.79 | 5403.58 | 5542.34 | .626 | 129, 89 | Selected |
| 3 | -2621.34 | 5366.67 | 5576.51 | .900 | 35, 14, 169 |  |
| 4 | -2596.58 | 5359.17 | 5640.08 | .916 | 14, 159, 32, 13 |  |
| 5 | -2572.27 | 5352.54 | 5704.53 | .796 | 71, 65, 9, 59, 14 |  |

**Supplementary Table 8B. Latent class descriptive comparison**

| **Variable** | **Class 1** | **Class 2** | **p** |
| --- | --- | --- | --- |
| Class size, n (%) | 89 (40.8%) | 129 (59.2%) |  |
| Total discordance score, mean ± SD | 4.0 ± 1.8 | 8.6 ± 2.4 | < .001 |
| Patient age, mean ± SD | 66.1 ± 12.3 | 63.0 ± 13.6 | .073 |
| Surrogate type: spouse, % | 50.6% | 55.0% | .375 |
| Surrogate type: child, % | 34.8% | 26.4% |  |
| Surrogate type: other, % | 14.6% | 18.6% |  |
| ACP discussion: discussed, % | 66.3% | 48.8% | .016 |
| Disease stage: early, % | 89.4% | 81.7% | .185 |
| Chemotherapy/targeted therapy: yes, % | 40.4% | 55.0% | .048 |

**Supplementary Figure 1. APIM path diagram for spouse dyads (n = 116 patients, 175 family members).**

**Solid lines with β coefficients denote significant paths (p < .05); dashed grey lines denote non-significant paths.*

**Supplementary Figure 2. APIM path diagram for child dyads (n = 114 patients, 196 family members).**

**Solid lines with β coefficients denote significant paths (p < .05); dashed grey lines denote non-significant paths.*

**Supplementary Figure 3. APIM path diagram for high-discordance dyads (n = 108; median split ≥ 6.5).**

**Solid lines with β coefficients denote significant paths (p < .05); dashed grey lines denote non-significant paths.*

**Supplementary Figure 4. APIM path diagram for low-discordance dyads (n = 109; median split < 6.5).**

**Solid lines with β coefficients denote significant paths (p < .05); dashed grey lines denote non-significant paths.*

**Supplementary Figure 5A. Latent class profiles of dyad discordance.**

**Heatmap of item-level discordance probabilities by latent class (2-class solution; BIC = 5542, entropy = 0.626).*

**Supplementary Figure 5B. Significant partner effects by latent discordance class.**

** Only significant cells are labelled with β and p.*
